# Facile NiO_x_ Sol-Gel Synthesis Depending on Chain Length of Various Solvents without Catalyst for Efficient Hole Charge Transfer in Perovskite Solar Cells

**DOI:** 10.3390/polym10111227

**Published:** 2018-11-06

**Authors:** Byung Gi Kim, Woongsik Jang, Dong Hwan Wang

**Affiliations:** School of Integrative Engineering, Chung-Ang University, 84 Heukseok-Ro, Dongjak-gu, Seoul 06974, Korea; ayct88@hanmail.net (B.G.K.); dndtlr2@cau.ac.kr (W.J.)

**Keywords:** nickel oxide sol-gel, facile organic solvent synthesis, perovskite solar cells, uniform morphology, electrical property

## Abstract

Nickel oxide (NiO_x_)–based perovskite solar cells (PSCs) have recently gained considerable interest, and exhibit above 20% photovoltaic efficiency. However, the reported syntheses of NiO_x_ sol-gel used toxic chemicals for the catalysts during synthesis, which resulted in a high-temperature annealing requirement to remove the organic catalysts (ligands). Herein, we report a facile “NiO_x_ sol-gel depending on the chain length of various solvents” method that eschews toxic catalysts, to confirm the effect of different types of organic solvents on NiO_x_ synthesis. The optimized conditions of the method resulted in better morphology and an increase in the crystallinity of the perovskite layer. Furthermore, the use of the optimized organic solvent improved the absorbance of the photoactive layer in the PSC device. To compare the electrical properties, a PSC was prepared with a p-i-n structure, and the optimized divalent alcohol-based NiO_x_ as the hole transport layer. This improved the charge transport compared with that for the typical 1,2-ethanediol (ethylene glycol) used in earlier studies. Finally, the optimized solvent-based NiO_x_ enhanced device performance by increasing the short-circuit current density (*J*_sc_), open-circuit voltage (*V*_oc_), and fill factor (*FF*), compared with those of poly(3,4-ethylenedioxythiophene):poly(styrenesulfonate)–based devices.

## 1. Introduction

Over the past few years, the photovoltaic R&D community has presented a breakthrough in the next generation of energy devices, along with numerous research achievements. This is the perovskite solar cell (PSC), which uses a halogenated perovskite (APbX_3_, *A* = monovalent cation and *X* = halide) [1]. These solar cells have a power conversion efficiency (PCE (%)) of ~22.7%; among them, the MAPbI_3_ (CH_3_NH_3_PbI_3_)–based solar cell has been reported to exhibit an excellent light conversion efficiency [2,3,4,5,6,7]. PSCs have two types of junctions: p-i-n [8,9,10,11,12,13,14] or n-i-p [15], in which the perovskite film is sandwiched between electrons (n) and holes (p). The n-i-p structure–based PSCs using a transparent electron transport layer (ETL) are the most common. Therefore, many studies have reported an n-i-p–type PSC configuration exhibiting the best performance [16,17]. However, studies on the inverted p-i-n junction, with a transparent hole transport layer (HTL), are also important. This is because of the following reasons: (a) high charge transfer between the inorganic HTL/perovskite interface [17,18,19,20], (b) suppression of hysteresis in the graph of JV characteristics [21,22] and (c) the cost effectiveness of the device [23,24,25,26,27].

On the other hand, the application of PSCs to determine the role of an inorganic material HTL having inherent photoelectric characteristics and high stability is emerging as an interesting subject [28,29,30,31,32]. Hence, we tried to optimize the synthesis of a spin-coated inorganic metal oxide, nickel oxide (NiO_x_), as an HTL applicable to inverted PSCs. It is important to control the stability of the sol, i.e., precipitation, in the synthesis of the sol-gel solution of the nickel precursor.

According to the literature, there are many cases in which bulky alcohols are used to prevent the precipitation of branched nickel precursors [33]. However, in the synthesis of the sol-gel solution, a rapid precipitation of the precursors has been observed in common alcoholic solvents such as ethanol and isopropanol. For example, the use of 2-methoxyethanol (methyl cellosolve) causes precipitation [32,34,35,36]. It has been observed that the use of the bulky 2-methoxyethanol solvent in the sol does not improve the solution stability at room temperature. Therefore, the precipitates of the nickel precursor could be dissolved only by increasing the temperature [37,38,39,40,41]. In earlier studies, toxic chemicals (monoethanolamine or ethylenediamine, as a typical chelating agent) have also been used to improve the solubility of the sol-gel solution. However, high-temperature annealing was inevitable, to remove the residual organic toxic catalysts in the solution, after synthesis [42,43,44,45]. 

Hence, a divalent alcohol–based solvent (having one more hydroxyl group than a monohydric alcohol–based solvent) was selected to synthesize the NiO_x_ sol-gel without a toxic catalyst. It was observed that the nickel precursor did not precipitate, and the stability of the solution improved [46,47,48,49,50]. We found that the results of this experiment were based on the evaporation rate effect of the boiling points of the different solvents [51,52,53,54,55,56,57,58,59]. Finally, we optimized the facile synthesis method of NiO_x_ sol-gels using dihydric alcohol solvents, such as 1,2-ethanediol (ET-OH), 1,4-butanediol (B-OH) and 1,5-pentanediol (P-OH) (the detailed synthesis procedure is described in the Experimental Section). NiO_x_ thin films were also prepared using these sol-gels, with three organic solvents having different bond lengths and strengths. Thus, we used the reproducible organic solvent–based synthesis of NiO_x_ sol-gels, and fabricated PSCs to identify the variation in the photovoltaic parameters. Unlike the previously reported solvents, the new solvent-based NiO_x_ was applied to an inverted PSC to clearly distinguish the PCE (%) changes. The optimized solvent-based NiO_x_ improved the device performance by increasing the short-circuit current density (*J*_sc_) and open-circuit voltage (*V*_oc_), compared with those of poly(3,4-ethylenedioxythiophene): poly(styrenesulfonate)(PEDOT:PSS)–based devices.

## 2. Materials and Methods 

### 2.1. Material Preparation

The NiO_x_ solution was synthesized by mixing a certain amount of Ni(NO_3_)_2_·6H_2_O precursor with 1,2-Ethylenediol, 1,4-Butanediol, and 1,5-Pentanediol (All chemicals of NiO_x_ were purchased from Sigma Aldrich, Saint Louis, MO, USA). For the other hole transport layer (HTL), poly (3,4 ethylenedioxythiophene):polystyrene sulfonate (PEDOT:PSS), AI 4083 was supplied by CLEVIOS™ (Heraeus, Suwon, Korea). For the MAPbI_3_ solution, MAI (CH_3_NH_3_I, Dyesol-Timo Co., Ltd., Seongnam, Gyeonggi-do, Korea) and 99.99% lead (II) iodide (TCI Co., Ltd., Taipei, Taiwan) were dissolved and stirred in a mixture of gamma-Butyrolactone (GBL), purchased from Sigma-Aldrich (Saint Louis, MO, USA), and dimethyl sulfoxide (DMSO), purchased from Junsei Co., Ltd. (Tokyo, Japan). All other chemicals were used from Sigma Aldrich.

### 2.2. NiO_x_ Sol-Gel Preparation

**Figure 1:**
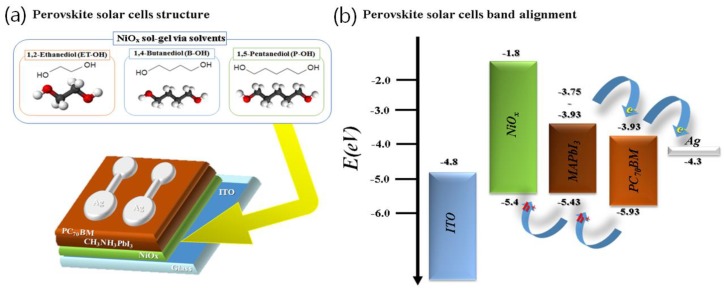
(**a**) A schematic diagram of an inverted planar perovskite solar cell using a hole transport layer with a NiO_x_ thin film via solvent. (**b**) Band energy alignment of the device.

Ni(NO_3_)_2_·6H_2_O precursor (Sigma-Aldrich, Saint Louis, MO, USA) was used for NiO_x_ sol-gel synthesis. The nickel precursor (1 M) was mixed with 10 mL of a divalent alcohol solvent of 1,2-ethylenediol (ET-OH), 1,4-butanediol (B-OH), and 1,5-pentanediol. The mixture was stirred at 75 °C for 2 h and then filtered through a 0.25 μm pore filter at room temperature. Three synthesized NiO_x_ sol-gels were slightly different in color depending on the solvent. The properties of the material are shown in Table S1, and the NiO_x_ solution and substrate photographs are shown in Figure S1. Using these materials, the device was fabricated with the composition shown in Figure 1 [60,61].

### 2.3. Thin Films and Device Fabrication

A patterned Indium tin oxide ITO substrate was first washed with solvents, DI water, acetone, and isopropanol and subsequently treated with O_2_-plasma for 15 min. A NiO_x_ film was spin-coated on the ITO substrate, at 1500 rpm for 60 s, and immediately transferred to a 120 °C hot plate and annealed in air at 300 °C for 1 h. After annealing, a MAPbI_3_ layer was deposited under an Ar environment. The MAPbI_3_ deposition was optimized by dissolving PbI_2_ and MAI solutes at a molar ratio of 1.06:1, a concentration of 1.20 M in Dimethylformamide (DMF):Dimethyl sulfoxide (DMSO), and a volume ratio 7:3.

### 2.4. Characterization of the Devices

The performances of the PSCs were measured using the PEC-L01 solar simulator (Peccell Technologies, Inc., Kanagawa, Japan), with an air-mass-1.5 global spectrum (AM 1.5 G) at an intensity of 100 mW/cm^2^. This spectrum was calibrated using a silicon reference cell. Furthermore, the current-density/voltage characteristics and impedance of the PSCs were analyzed using the ZIVE SP1 electrical measurement system (WonATech Co., Seoul, Korea). The device area was confirmed to be 0.118 cm^2^, based on the deposited Ag cathode of the mask pattern. The power calibration was measured using an LS150 device (Abet Technologies, Inc., Milford, CT, USA). The incident photon-to-current efficiency (IPCE) was determined using a MonoRa-500i monochromator (DongWoo Optron Co. Ltd., Gwangju, Gyeonggi-do, Korea). The surface morphology of the various thin-film layers were observed using a SIGMA-model scanning electron microscope SEM instrument (Carl Zeiss Inc., San Diego, CA, USA), at 5 kV. The crystallinity of the perovskite layer was analyzed by recording X-ray diffraction (XRD) spectra, using a New D8-Advance (Bruker, Seongnam, Gyeonggi-do, Korea) X-ray diffractometer. Photoluminescence (PL) spectroscopy analysis was performed using a micro-Raman instrument (XperRam 200, Nanobase, Guemcheon-Ku, Korea), with a 550 nm excitation and an incident power of 0.3 mW. Furthermore, the time-resolved photoluminescence (TRPL) was analyzed using a time-correlated single photon counting instrument, with a 454 nm excitation laser and an incident power of 0.3 µW, 25 times the average, 500 ms of exposure time, and 100 ps of resolution. The contact angle of water droplets on “glass/NiO_x_ via solvent (ET-OH, B-OH, and P-OH)” were measured with a Phoenix-150 instrument (SEO, Suwon-si, Gyeonggi-do, Korea).

## 3. Results and Discussion

The two key functions of our device relate to the device fabrication process (Figure 1a,b and Figure S1, and Experimental Section): (i) divalent organic acid–based NiO_x_ sol-gel synthesis without a catalyst and (ii) thin-film manufacturing process for optimizing the “NiO_x_ via solvent/MAPbI_3_” interfacial contact.

**Figure 2:**
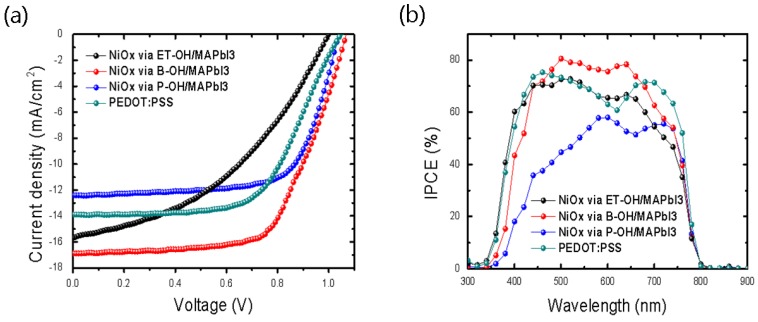
(**a**) J-V characteristics of inverted PSCs (Perovskite Solar Cells) using sol-gel-based NiO_x_ transport layers synthesized via three solvents and using PEDOTP:PSS (poly (3,4 ethylenedioxythiophene):polystyrene sulfonate); AM 1.5G, 100 mW/cm^2^ under solar simulaton. (**b**) IPCE (The incident photon-to-current efficiency) curves of the planar inverted PSC structure.

Figure 2a shows that the NiO_x_ HTLs (precursor sol, 1 mol/L) prepared with the divalent alcohol–based solvent led to a difference in the JV curve of the PSCs. When the photovoltaic performance analysis (Table 1) was taken into consideration, the *V*_oc_ of the devices were ~999–1069 mV after 10 min of light-irradiation. The change in the *V*_oc_, showing the intrinsic property of the interface quality, was not as high as the ~70 mV for the comparative group. Therefore, the difference in the *V*_oc_ was not observed to be greatly dependent on the solvent selectivity. On the other hand, a change in the PCE (%) was confirmed by the large differences in the *J*_sc_ and fill factor (*FF*). When the PCE (%) was 11.22% and the *FF* was 62%, the optimized cell corresponded with the NiO_x_ sol-gel synthesized via B-OH. When NiO_x_ via ET-OH was used, the *J*_sc_ was found to be 1.21 mA/cm^2^ lower than when the B-OH solvent was used; thus, the *J*_sc_ was found to depend on the type of sol-gel solvent.

As shown in Figure 2b, the highest IPCE value in the range of 400–650 nm and the integrated *J*_sc_ (same as *J*_sc,IPCE_) of 16.79 mA/cm^2^ were confirmed for the “NiO_x_ via B-OH”-based devices, compared with those of the others. The films of “NiO_x_ via solvent” had a similar curvilinear shape for the UV-Vis absorbance spectra (Figure S2a), which signified that the absorbance of the “NiO_x_ via B-OH/MAPbI_3_” thin film was higher in the range of 400–650 nm than that of the comparative group (Figure S2b).

**Figure 3:**
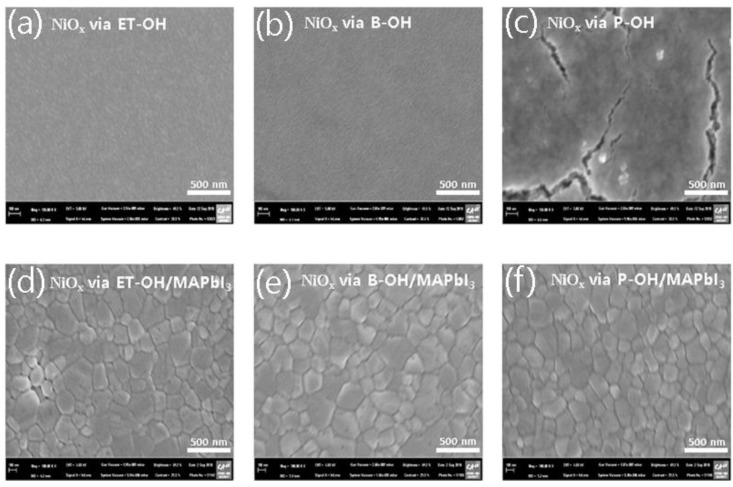
FE-SEM of ITO/NiO_x_ (sol-gel) via solvent: (**a**) ET-OH, (**b**) B-OH, (**c**) P-OH and MAPbI_3_ on ITO/NiO_x_ (sol-gel) via solvent, (**d**) ET-OH, (**e**) B-OH, and (**f**) P-OH.

Field emission scanning electron microscope (FE-SEM) analysis was performed to investigate the difference in morphology among the sol-gel solutions synthesized with divalent alcohol solvents (Figure 3). In the case of “NiO_x_ via P-OH” (Figure 3c), the NiO_x_ sol-gel was formed to a large extent, whereas the rest (Figure 3a,b) had a very flat and uniform substrate [62,63,64]. It is known that a smooth and uniform morphology can prevent the aggregation of precursors (accumulation of large amounts of perovskite) [65,66,67,68]. In fact, in the case of “NiO_x_ via B-OH” devices, it was confirmed that the performance of the device was optimal due to the homogeneity of NiO_x_. This is because a thin HTL with a uniform coverage of the substrate generally has a low series resistance and an improved shunt resistance [69,70,71].

As shown in Figure S4, contact angle analysis was performed to compare the NiO_x_ interfacial state before the deposition of the photoactive layer. The contact angle (Figure S4b) of the “NiO_x_ via B-OH” thin film (14.42°) was lower than that of the comparative group (Figure S4a,c). This confirmed that the state of this interface was relatively hydrophilic. This result indicates that the “NiO_x_ via B-OH” substrate is advantageous for interfacial adhesion when a photoactive layer (MAPbI_3_) is deposited by spin coating [72,73]. We also confirmed the successful casting of the MAPbI_3_ layer on the metal oxide, using the image of the film coverage of NiO_x_/MAPbI_3_ (Figure 3d–f).

**Figure 4:**
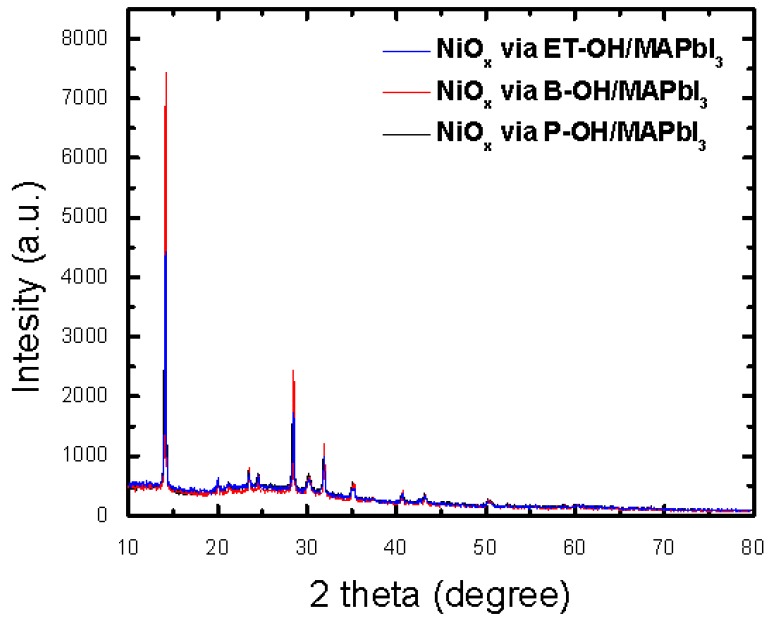
XRD patterns of MAPbI_3_ on ITO/NiO_x_ substrate via solvent (ET-OH, B-OH, and P-OH).

For the quantitative analysis of the previously analyzed nanoporous morphology trends, NiO_x_ sol-gel substrates were prepared as samples and subjected to XRD analysis (Figure S3). A relatively low full width at half maximum FWHM of 0.248 and nanoporous crystallite size of 373.5 Å were observed for NiO_x_ via B-OH, compared with those of the comparative group (Table 2). As shown in Figure 4, the XRD spectra of the samples deposited with the photoactive layer showed the sharpest intensity, in the case of the “NiO_x_ via B-OH” samples. This sample showed the lowest FWHM of 0.103, and the largest crystallite size of 860.6 Å. Therefore, we found that the relatively large grain size of the “NiO_x_ via B-OH” device was favorable for light absorption, in the comparative group. These results correlate with the higher values of the *J*_sc_. This is because the perovskite grain acts as a light-absorber, and its absorption depends on the size [74,75,76,77].

**Figure 5:**
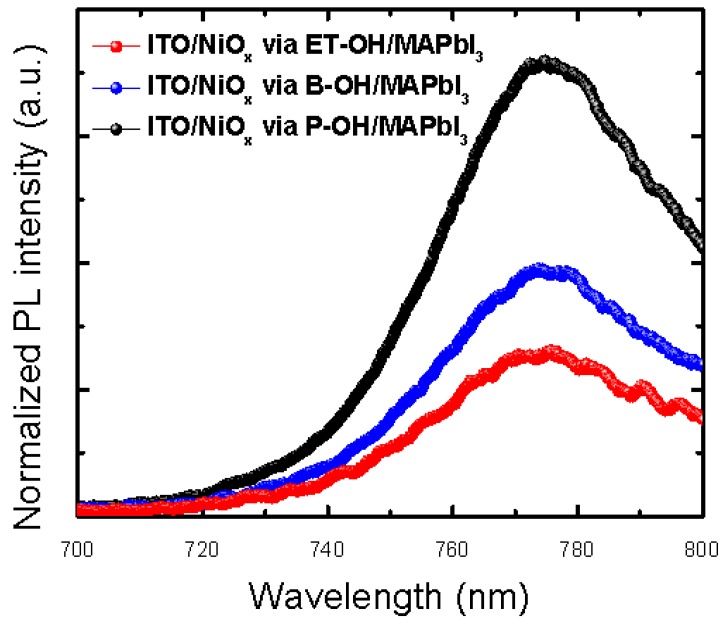
Normalized photoluminescence spectra of the glass/NiO_x_ (sol-gel) via solvent (ET-OH, B-OH, and P-OH)/perovskite(MAPbI_3_) film under 550 nm light excitation respectively.

In addition, electrical property analysis was performed to compare the exciton dissociation (or charge separation) differences. PL analysis was performed with different compositions of the “ITO/NiO_x_ via solvent (ET-OH, B-OH, and P-OH)/MAPbI_3_” samples (Figure 5). We found that the normalized PL intensity was the lowest in the “NiO_x_ via B-OH” samples. This suggests that the exciton dissociation (or charge separation) tendency of this material is superior [78,79,80,81]. It was also found that the PCE (%) values of the devices were consistent with this tendency (Table 1).

**Figure 6:**
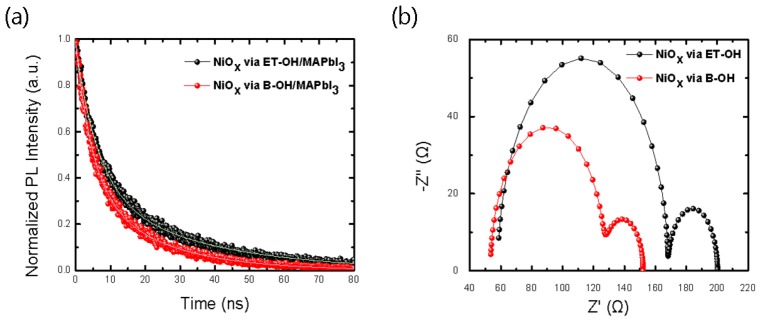
(**a**) Time-resolved transient PL spectra of glass/NiO_x_ via solvent (ET-OH and B-OH)/MAPbI_3_ substrate. The steady-state PL spectra were excited at a wavelength of 454 nm. (**b**) Dark impedance measurements of NiO_x_ via solvent (ET-OH and B-OH) based perovskite solar cells at Voc, DC bias. The dark DC bias is applied to obtain the most efficient charge extraction results with the smallest charge transport resistance in a NiO_x_ via B-OH based device.

Furthermore, in order to compare the electrical properties of the two solvents (ET-OH and B-OH), which resulted in a homogeneous and smooth morphology of the NiO_x_ thin films, we conducted TRPL analysis of these films, and impedance analysis, through the fabrication of PSCs (Figure 6). The results (Figure 6, Table 3) of the NiO_x_ via solvent (ET-OH and B-OH) show that the *τ*_1_, with a charge carrier quenching tendency, was shorter in the B-OH–applied sample, by 1.75 ns. On the other hand, the *τ*_2_ was longer in the ET-OH–applied sample, by 11.5 ns, with a charge-recombination tendency [82,83,84]. In conclusion, we found a relatively better charge transfer (or charge separation) tendency in the B-OH materials than that of the ET-OH materials, which were commonly used in earlier studies [60,61], because the average *τ*_avg._ value was shorter in the B-OH samples by 8.83 ns [85,86,87]. 

As shown in Figure 6b and Table 3, the impedance results were analyzed using a PSC. This was carried out with reference to the circuit diagram in an earlier study [88]. The series resistances (*R*_s_, charge transport resistance) were 7.45 × 10^1^ Ω (*R*_s1_) and 2.43 × 10^1^ Ω (*R*_s2_), which were lower in the “NiO_x_ via B-OH” devices than those in the “NiO_x_ via ET-OH” devices. Therefore, this is consistent with the device performance results, which show an improved *J*_sc_ and PCE (%), as shown in Table 1 [89,90,91].

Figure S5a,b shows a stable device operation over 1200 h and relatively high durability was confirmed in NiO_x_ via B-OH based cell. Additionally, statistical analysis of device performance showed that the average PCE (%) was superior to that of the comparative group. (Table S2). Thus, it was confirmed that solvent selectivity is important in NiO_x_ sol-gel synthesis, based on the device performance data. The device also showed a PCE (%) difference of 1.48% compared with that for PEDOT:PSS (Table 1), indicating that it can be used as a material comparable to PEDOT:PSS.

## 4. Conclusions

Catalyst (stabilizer)-free and reproducible NiO_x_ sol-gels were synthesized by selecting three organic solvents with different chain lengths of dihydric alcohols (hydroxyl groups, -diols). A transparent ITO/NiO_x_ substrate was applied as an HTL, using the NiO_x_ sol-gels to fabricate inverted PSCs. The interfacial contact of NiO_x_/MAPbI_3_ and the electrical characterization results showed significantly improved properties in NiO_x_ via B-OH, compared with those of the comparative group. Therefore, this study shows a new effect of NiO_x_ thin film depending on the chain length of various solvents for efficient hole charge transfer on PSCs without catalyst. Furthermore, this study has shown that the optimized NiO_x_ material can be used as an HTL in place of acidic PEDOT:PSS, which is expected to be a promising stepping stone in the study of metal oxide interlayers.

## Figures and Tables

**Table 1:** Performance parameters of the planar inverted PSC devices.

HTLs/Parameter	*V*_oc_	*J*_sc_	*J*_sc_IPCE_	*FF*	PCE%
NiO_x_ via ET-OH	0.999	15.68	15.41	0.42	6.56
NiO_x_ via B-OH	1.069	16.89	16.79	0.62	11.22
NiO_x_ via P-OH	1.039	12.42	11.78	0.69	8.91
PEDOT:PSS	0.969	16.18	16.53	0.62	9.74

**Table 2:** Crystallite size with FWHM (Full Width at Half Maximum) on ITO/NiO_x_ via solvents and ITO/NiO_x_ via solvents/MAPbI_3_ substrate.

Sample Condition	FWHM	C. Size (Å)
ITO/NiO_x_ via ET-OH	0.285	324.8
ITO/NiO_x_ via B-OH	0.248	373.5
ITO/NiO_x_ via P-OH	0.258	358.8
NiO_x_ via ET-OH/MAPbI_3_	0.252	353.6
NiO_x_ via B-OH/MAPbI_3_	0.103	860.6
NiO_x_ via P-OH/MAPbI_3_	0.167	533.6

**Table 3:** Parameters of the TRPL (Time-Resolved Photo Luminescence) spectra and impedance.

NiO_x_ via Solvent	*τ*_1_ (ns)	*τ*_2_ (ns)	*τ*_avg_ (ns)	Fraction 1 (%)	Fraction 2 (%)	*R*_s1_ (Ω)	*R*_s2_ (Ω)
ET-OH	6.42	31.91	24.9	0.72	0.38	1.10 × 10^2^	3.20 × 10^1^
B-OH	4.67	20.41	16.07	0.68	0.41	7.45 × 10^1^	2.43 × 10^1^

## Supplementary Materials

The following are available online at http://www.mdpi.com/2073-4360/10/11/1227/s1, Figure S1: photographs of NiO_x_ via solvents: (a) ET-OH, (b) B-OH, and (c) P-OH sol-gel solutions and ITO/NiO_x_ via solvents, (d) ET-OH, (e) B-OH, and (f) P-OH substrates, Figure S2: absorbance spectra of (a) ITO/NiO_x_ via solvents (ET-OH, B-OH, and P-OH) and (b) glass/NiO_x_ via solvents (ET-OH, B-OH, and P-OH)/MAPbI_3_ based on air reference, Figure S3: XRD patterns of glass/ITO/NiO_x_ via solvents (ET-OH, B-OH, and P-OH), Figure S4: the contact angle images of water droplet (H_2_O) on different surfaces: (a) glass /NiO_x_ via ET-OH, (b) glass/NiO_x_ via B-OH, and (c) glass /NiO_x_ via P-OH, Figure S5: (a) histogram of PCE (%) device performance for 20 perovskite solar cells fabricated under NiO_x_ via solvent control, (b) Normalized PCE (%) of a perovskite solar cell containing HTLs of NiO_x_ via ET-OH (black) and B-OH (red) measured under ambient environmental conditions and standard AM 1.5 solar illumination, Table S1: basic properties of the three solvents used in NiO_x_ sol-gel synthesis, Table S2: PCE (%) statistical data of perovskite solar cells based on NiO_x_ via solvent.
