# Supplementary material for: Facile NiOx Sol-Gel Synthesis Depending on Chain Length of Various Solvents without Catalyst for Efficient Hole Charge Transfer in Perovskite Solar Cells
Source: Polymers (Basel). 2018 Nov 6;10(11):1227. doi: 10.3390/polym10111227 (PMC6290588; doi:10.3390/polym10111227)
Supplement: Supplementary file 1 [file polymers-10-01227-s001.pdf]

(Supplementary Information)

Facile NiO_x sol-gel synthesis depending on chain length of various solvents without catalyst for efficient hole charge transfer in perovskite solar cells

Byung Gi Kim, Woongsik Jang, and Dong Hwan Wang*

Table S1. Basic properties of the three solvents used in NiO_x sol-gel synthesis.

Solvents	Density (g/cm^3)	Boiling point (°C)	Molecular weight (g/mol)	Viscosity (Pa·s)	Chemical formula
1,2-Ethanediol (ET-OH)	1.11	197.3	62.07	1.61×10^{-2}	$C_2H_6O_2$
1,4-Butanediol (B-OH)	1.02	230	90.12	84.9×10^{-3}	$C_4H_{10}O_2$
1,5-Pentanediol (P-OH)	0.994	242	104.15	128×10^{-3}	$C_5H_{12}O_2$

Table S2. PCE (%) statistical data of perovskite solar cells based on NiO_x via solvent.

Condition	Maximum PCE (%)	Minimum PCE (%)	Average PCE (%) of 20 unit cells
NiO_x via ET-OH	9.52	6.08	8.45
NiO_x via B-OH	11.74	10.50	11.14
NiO_x via P-OH	10.58	7.13	8.97

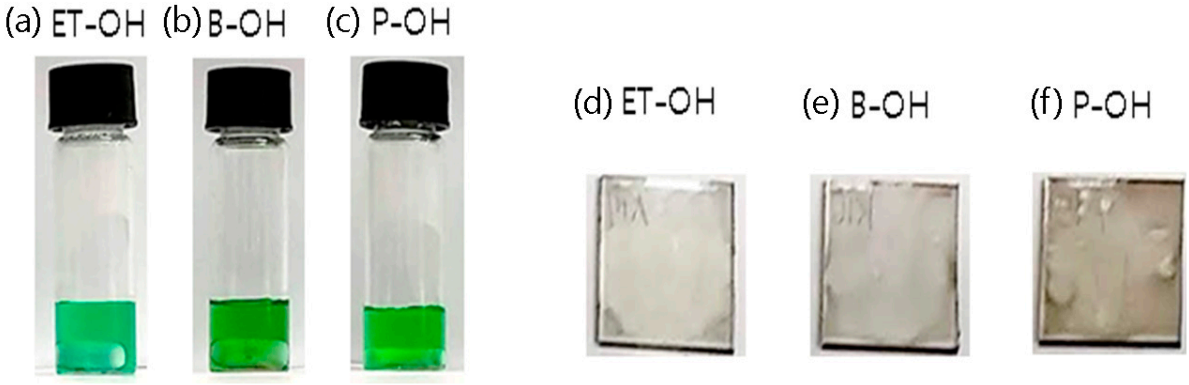

Figure S1. Photographs of NiO_x via solvents (**(a)** ET-OH, **(b)** B-OH, and **(c)** P-OH) sol-gel solutions and ITO/ NiO_x via solvents (**(d)** ET-OH, **(e)** B-OH, and **(f)** P-OH) substrates.

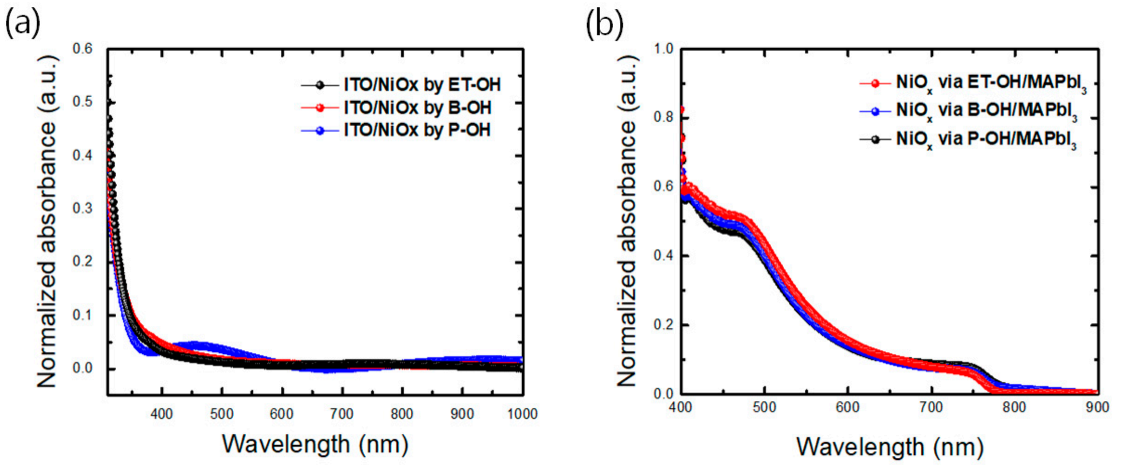

Figure S2. Absorbance spectra of **(a)** ITO/NiO_x via solvents (ET-OH, B-OH, and P-OH) and **(b)** glass/NiO_x via solvents (ET-OH, B-OH, and P-OH)/$MAPbI_3$ based on air reference.

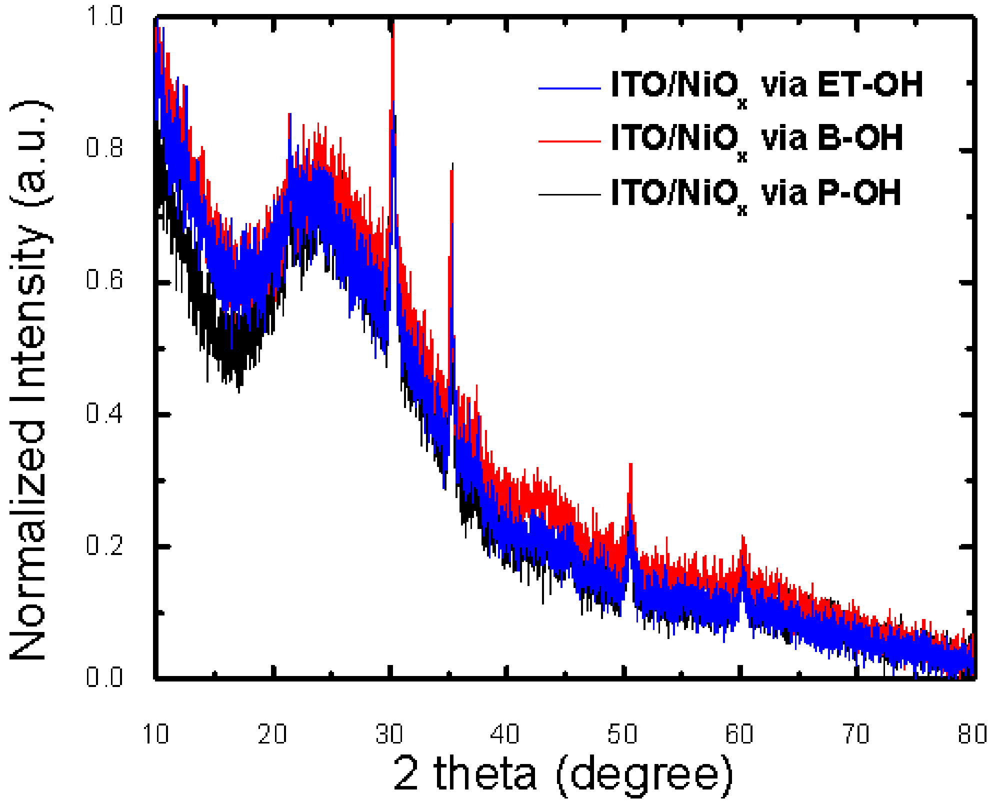

Figure S3. XRD patterns of glass/ITO/NiO_x via solvents (ET-OH, B-OH, and P-OH).

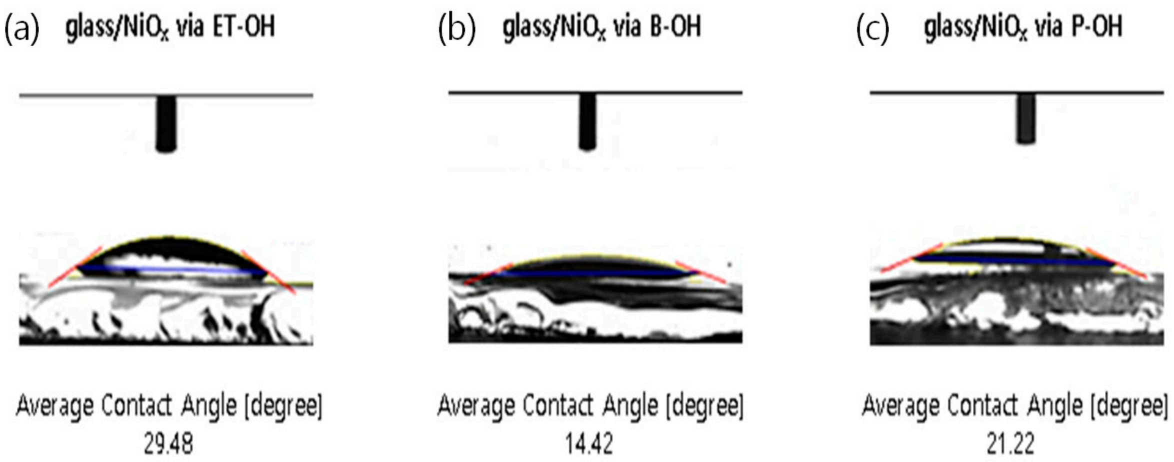

Figure S4. The contact angle Images of water droplet (H_2O) on different surfaces; **(a)** glass /NiO_x via ET-OH, **(b)** glass /NiO_x via B-OH, and **(c)** glass /NiO_x via P-OH.

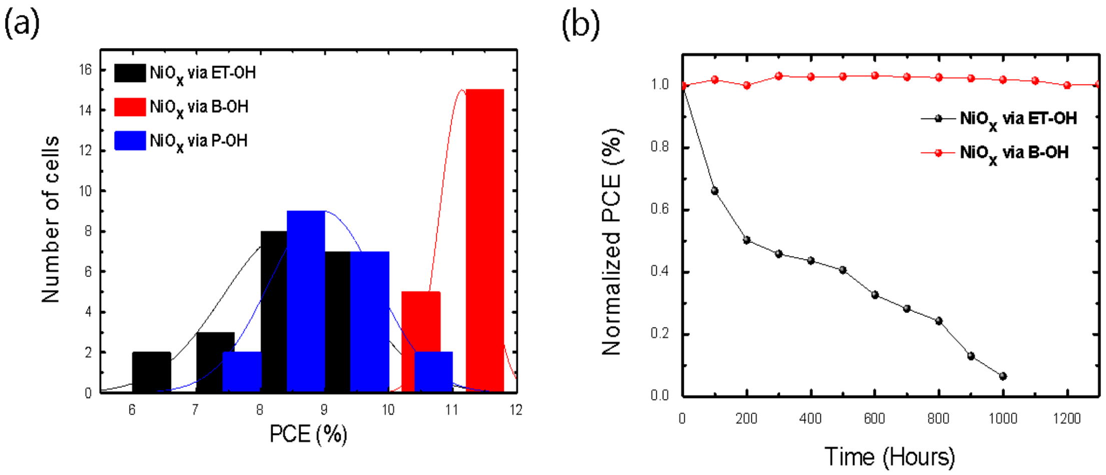

Figure S5. (a) Histogram of PCE (%) device performance for 20 perovskite solar cells fabricated under NiO_x via solvent control **(b)** Normalized PCE (%) of a perovskite solar cell containing HTLs of NiO_x via ET-OH (black) and B-OH (red) measured under ambient environmental conditions and standard AM 1.5 solar illumination.
